# Nutritional Evaluation of Commercially Important Fish Species of Lakshadweep Archipelago, India

**DOI:** 10.1371/journal.pone.0045439

**Published:** 2012-09-21

**Authors:** Kottila Veettil Dhaneesh, Kunnamgalam Mohammed Noushad, Thipramalai Thankappan Ajith Kumar

**Affiliations:** 1 Centre of Advanced Study in Marine Biology, Faculty of Marine Sciences, Annamalai University, Parangipettai, Tamil Nadu, India; 2 Centre for Marine Living Resources and Ecology, Ministry of Earth Sciences, Field Research Station, Agatti Island, U. T. of Lakshadweep, India; University of Sao Paulo, Brazil

## Abstract

Estimation of nutrition profile of edible fishes is essential and thus a bio-monitoring study was carried out to find out the nutritional composition of commonly available fishes in Agatti Island water of Lakshadweep Sea. Protein, carbohydrate, lipid, ash, vitamin, amino acid and fatty acid composition in the muscle of ten edible fish species were studied. Proximate analysis revealed that the protein, carbohydrate, lipid and ash contents were high in *Thunnus albacares* (13.69%), *Parupeneus bifasciatus* (6.12%), *Hyporhamphus dussumieri* (6.97%) and *T. albacares* (1.65%), respectively. Major amino acids were lysine, leucine and methionine, registering 2.84–4.56%, 2.67–4.18% and 2.64–3.91%, respectively. Fatty acid compositions ranged from 31.63% to 38.97% saturated (SFA), 21.99–26.30% monounsaturated (MUFAs), 30.32–35.11% polyunsaturated acids (PUFAs) and 2.86–7.79% branched fatty acids of the total fatty acids. The ω-3 and ω-6 PUFAs were ranged 13.05–21.14% and 6.88–9.82% of the total fatty acids, respectively. Hence, the fishes of Lakshadweep Sea are highly recommended for consumption, since these fishes are highly enriched with nutrition. The results can be used as a baseline data for comparing the various nutritional profiles of fishes in future.

## Introduction

Fish is a major source of food for mankind, providing with a significant amount of the animal protein diet, excellent dietary sources of highly unsaturated fatty acid (HUFA) and polyunsaturated fatty acid (PUFA), especially the omega-3 fatty acids, eicosapentaenoic acid (EPA) and docosahexaenoic acid (DHA) [1]. Today, there is increasing interest in fish consumption because of their high PUFA content. Moreover, consumption of fish has been linked to health benefits, as the long chain PUFA has gained attention because of prevention of human coronary artery disease [2], improvement of retina and brain development [3], decreased incidence of breast cancer, rheumatoid arthritis, multiple scelerosis, asthma, psoriasis, inflammatory bowel disease [4], [5] and regulation of prostaglandin synthesis [6].

Extensive research has been done on fish lipids, fatty acids [7] and amino acids. However, a big variation has been noticed in these compositions of different individuals of the same fish species. Researchers have found that freshwater fish contain lower proportions of ω-3 PUFA than the marine fish [8]. Therefore, the ratio of total ω3–ω6 fatty acids is much higher in the marine fish than for freshwater fish, varying from 5 to 10 times or more. Nutritionists believe that the desirable ratio of ω6/ω3 should be 5 and the addition of ω3 polyunsaturated fatty acids (ω3 PUFA) could improve the nutritional value and prevent diseases [9]. Fatty acid composition may also be affected by environmental factors [10] or size or age of the animals [11], affecting metabolic activity. Certain amino acids like aspartic acid, glycine and glutamic acid are also known to play a key role in the process of wound healing [12]. Therefore, when fish is suggested for consumption, both fat content and the PUFA composition must be considered. Although it is generally recognised that PUFA composition may vary among species, little attention has been paid to the nutritional composition of different fish species while selecting for diet. As the Lakshadweep Sea is vastly supplied with a great variety of fish species, islanders are highly dependent on fish for food. However, knowledge concerning the nutritional quality of the commercially important fishes of India [13], [14] especially from the Lakshadweep Sea is limited. So, this study was carried out to determine the fatty acid, amino acid, vitamin, protein, carbohydrate, lipid and ash content of ten edible fishes, most commonly consumed by the local population of the Lakshadweep Island.

## Materials and Methods

### 2.1 Study area

Agatti Island lies in the Lakshadweep group of islands (latitude 10°48′–10°52′N; longitude 72°10′–72°12′E) in the Lakshadweep Sea of the Indian Ocean which is a part of Chagos - Maldives - Lakshadweep archipelago. Among the 425 atolls of the world, it is the largest atoll system with 12 atolls. This atoll system has grown up steeply from a depth about 1,500 to 4,000 m over the Chagos - Laccadive oceanic ridge. Lakshadweep consists of 36 islands covering an area of 32 sq. km of which 10 are inhabited. These islands lie scattered in the Lakshadweep Sea about 225 to 445 km from the main land coast. The islands are about 1–2 meters above the sea level. It has a total lagoon area of about 4, 200 sq. km, territorial water area of 20, 000 sq. km and an exclusive economic zone of 4, 00,000 sq. km

### 2.2 Sample collection

Ten marine fish species (*Epinephelus tauvina, Carangoides orthogrammus, Tylosurus crocodilus crocodiles, Lutjanus gibbus, Seriola lalandi, Thunnus albacares, Parupeneus bifasciatus, Chelinus undulates, L. bohar, Hyporhamphus dussumieri*) were collected from the Lakshadweep Sea, during October, 2011. Total fish lengths were measured and significant differences were found among different species (*F*_(9, 10)_ = 670.83, *P*<0.05). After collection, samples were kept in plastic bags and transported in an insulated icebox to the laboratory and were beheaded, gutted, washed and filleted.

### 2.3 Determination of total protein

Folin - Ciocalteau Phenol method of Lowry et al. [15] was used for the determination of the total protein in the tissue. The dried tissue sample weighing 10±0.1 mg was thoroughly homogenised with 1 mL of deproteinising agent (10% TCA) by keeping the tube in ice. All samples were centrifuged for 20 min at 3000 rpm. The precipitate obtained was used for protein estimation. The precipitate was dissolved in 2 mL 1N NaOH and to 1 mL of this solution, freshly prepared 5 mL alkaline reagent was added. This was kept at room temperature for 10 min, after which 0.5 mL of 1N Folin - Ciocalteu reagent (Hi-media, India) was added and mixed rapidly. A standard solution was prepared by using Bovine serum albumin (Hi-media, India) crystal at a concentration of 0.2 mg/mL from the stock solution. A blank was prepared with 1 mL 1N NaOH and treated the same way as above. All the test tubes were kept for 30 min at room temperature in dark and the optical density (OD) of the blue colour developed was measured against the blank at 660 nm (Shimadzu UV-1800 UV spectrophotometer, Japan).

### 2.4 Determination of total carbohydrate, lipid and ash

Total carbohydrate was estimated by Phenol-Sulphuric acid method, described by Dubois et al. [16]. About 5±0.1 mg of oven-dried tissue was taken in a test tube and 1 mL of phenol (5%) and 5 mL of concentrated sulphuric acid were added in quick succession. The tube was kept for 30 min at 30°C and the optical density of the colour developed was measured at 490 nm against the blank (Shimadzu UV-1800 UV spectrophotometer, Japan).

Lipid content was estimated by the procedure given by Folch et al. [17]. About 500±0.1 mg of powdered oven dried tissue was mixed with 5 mL of chloroform∶ methanol (2∶1) mixture tightly covered with aluminium foil and kept at room temperature for 24 h. It was then filtered by using Whatman No. 1 filter paper (11 µm) and the filtered extract was taken in a pre-weighed beaker and oven dried. Beaker was weighed with lipids and the difference in weight was taken as total lipid content and percentage was calculated. Ash content was determined gravimetrically by incinerating 1±0.01 g dried sample in Muffle furnace at about 550°C for 6 h and the results are expressed in percentage [18].

### 2.5 Estimation of vitamins

Fat soluble vitamins A, D3 and K and the water soluble vitamins B1, B2 and B6 were analysed in the HPLC (Merck - Hitachi L - 7400), following the method described by Sadasivam and Manickam [19]. The folic acid was estimated adopting the calorimetric procedure of Sethi [20]. Vitamin B5 was estimated, as suggested in USP NF 2000 Asian edition.

### 2.6 Amino acid analysis

Powdered oven dried samples were extracted well and hydrolysed in 6 N HCl at 110°C for 24 h and the amino acids were estimated in the HPLC (Merck - Hitachi L - 7400) following the method of Baker and Han [21].

### 2.7 Fatty acid analysis

For fatty acid analysis, each sample was oven dried at 67°C for 24 h and ground finely with mortar and pestle. The preparation and analysis of fatty acid methyl esters (FAMEs) from the fish tissues were performed according to the method described by Anon [22]. About 50±0.1 mg of tissue samples were added to 1 mL of 1.2 M NaOH in 50% aqueous methanol with glass beads (3 mm dia) in a screw-cap tube and then incubated at 100°C for 30 min in a water bath. The saponified samples were cooled at room temperature for 25 min, they were acidified and methylated by adding 2 mL 54% 6 N HCl in 46% aqueous methanol and incubated at 80°C for 10 min in water bath. After rapid cooling, methylated FAs were extracted with 1.25 mL 50% diethyl ether in hexane. Each sample was mixed for 10 min and the bottom phase was removed with a pasteur pipette. Top phase was washed with 3 mL 0.3 M NaOH. After mixing for 5 min, the top phase was removed for analysis. Following the base wash step, the FAMEs were cleaned in anhydrous sodium sulphate and then transferred to GC sample vial for analysis. FAMEs were separated by gas chromatograph (HP 6890 N, Agilent Technologies, USA). FAMEs profiles of the tissues were identified by comparing the commercial Eucary data base with MIS Software package (MIS Ver. No. 3.8, Microbial ID. Inc., Newark, Delaware).

### 2.8 Statistical analysis

Pearson Correlation Coefficient was employed for the better understanding of relationship between the percentage of protein, carbohydrate, lipid, ash, vitamins, amino acids and fatty acids with various fish species, using the statistical package of SPSS 16.0. One way ANOVA was also employed to understand the variation in the quantity of nutrients with respect to different species.

## Results and Discussion

### 3.1 Proximate composition

Total protein, total lipid, total carbohydrate and total ash contents of the muscle of ten fishes of Lakshadweep Sea are shown in Table 1. The lipid content of fish differed which could be due to variation of species, diet, geographical origin, age and season [23]. In this study, lipid content ranged from 2.96% (*T. albacares*) to 6.97% (*H. dussumieri*) which could be classified as lean or semi-fatty fish. The crude lipid content was higher than the content (4.95%) found in pomfret, *Pampus punctatissimus* [24]. According to Ozogul and Ozogul [25], fatty fish usually contain a minimum of 5–8% fat in edible tissue. Low-fat fish have higher water content; as a result, their flesh is white in colour [26]. Fatty fish store the fat in muscle tissue and so their flesh colour is yellow, grey and pink [27]. Higher total protein content (13.69%), total carbohydrate (6.12%) and total ash (1.65%) were found in *T. albacares*, *P. bifasciatus* and *L. bohar* respectively. Lower total protein (10.51%), total carbohydrate (2.97%) and total ash (1.05%) were found in *H. dussumieri*, *L. gibbus* and *E. tauvina*, respectively. Significant positive correlation was recorded by protein with ash (*r* = 0.271) but negatively correlated with carbohydrate (*r* = −0.031) and lipid (*r* = −0.765, *P*<0.05). Carbohydrate is positively correlated with lipid (*r* = 0.231) and ash (*r* = 0.431) while lipid was negatively correlated with protein (*r* = −0.765, *P*<0.05) and ash (*r* = −0.258). In case of ash, it was positively correlated with protein (*r* = 0.271) and carbohydrate (*r* = 0.431) but negatively correlated with lipid (*r* = −0.258). Proximate composition values varied significantly with respect to various fish species (*F*_(3, 32)_ = 153.582, *P*<0.05).

**Table 1:** Proximate composition of ten fish species from Lakshadweep Sea.

Species	Crude Protein(% dry weight)	Crude Carbohydrate(% dry weight)	Crude Lipid(% dry weight)	Crude Ash(% dry weight)
***E. tauvina***	11.54	4.56	3.55	1.05
***C. orthogrammus***	11.51	3.92	4.21	1.51
***T. crocodilus crocodilus***	13.26	3.68	4.56	1.39
***L. gibbus***	10.58	2.97	4.78	1.28
***S. lalandi***	12.53	4.23	3.11	1.11
***T. albacares***	13.69	5.56	2.96	1.57
***P. bifasciatus***	10.58	6.12	5.56	1.56
***C. undulates***	10.54	4.16	5.28	1.19
***L. bohar***	11.67	5.91	4.54	1.65
***H. dussumieri***	10.51	5.68	6.97	1.14

### 3.2 Vitamins

Vitamins present in the fishes are shown in Table 2. Among the fishes, *H. dussumieri* showed higher content of vitamins (5.29 mg per 100 gm) and *E. tauvina* showed comparatively the least (4.01 mg per 100 gm). Vitamin values did not show significant variations with respect to various fish species (*F*_(9, 80)_ = 0.4). Vitamins present in *E. tauvina* was positively correlated with that of *H. dussumieri* (*r* = 0.766, *P*<0.05), while that of *L. gibbus* was positively correlated with *C. undulates* (*r* = 0.814, *P*<0.01)

**Table 2:** Vitamins (mg/100 gm) of ten fish species from Lakshadweep Sea.

Vitamins	*E. tauvina*	*C. orthogrammus*	*T. crocodilus crocodilus*	*L. gibbus*	*S. lalandi*	*T. albacares*	*P. bifasciatus*	*C. undulates*	*L. bohar*	*H. dussumieri*
**Vitamin B1**	0.44	0.42	0.39	0.48	0.56	0.61	0.54	0.44	0.48	0.39
**Vitamin B2**	0.65	0.13	0.48	0.56	0.67	0.59	0.44	0.48	0.37	0.86
**Vitamin B3**	0.22	0.31	0.41	0.52	0.49	0.37	0.19	0.61	0.87	0.51
**Vitamin B5**	0.41	0.81	0.31	0.44	0.71	0.27	0.53	0.19	0.18	0.37
**Vitamin B6**	0.11	0.19	0.34	0.58	0.41	0.49	0.67	0.56	0.91	0.43
**Folic acid**	0.81	0.92	0.79	0.84	0.81	0.77	0.63	0.92	0.55	0.84
**Vitamin A**	0.55	0.62	0.72	0.33	0.19	0.61	0.62	0.33	0.49	0.68
**Vitamin K**	0.43	0.65	0.19	0.41	0.65	0.36	0.41	0.55	0.53	0.67
**Vitamin D3**	0.39	0.51	0.44	0.51	0.37	0.77	0.51	0.62	0.42	0.54

### 3.3 Amino acid

Amino acid composition in the muscle of ten fishes is shown in Table 3. It was found that among the fishes, quantity of essential amino acids, histidine (3.17%), threonine (2.92%) and valine (2.76%) was higher in *H. dussumieri*, followed by *T. albacares* which showed higher amount of lysine (4.56%) and phenylalanine (3.77%). Lysine is the limiting amino acid in cereal-based diets of children in developing countries [28]. Lysine is the highly accumulated essential amino acid (4.56%) in muscles of *T. albacares* and cystine, the non essential amino acid (2.55%) in *L. gibbus*. Among the non essential amino acids, asparagine (1.92%), cystine (2.55%) and tyrosine (0.94%) were accumulated in higher amounts in *L. gibbus* followed by *S. lalandi* (Glutamic acid 2.17% and Glycine 1.84%). According to Zhao et al. [24], amino acid composition of pomfret muscle showed a higher amount of glutamic acid followed by lysine, leucine, aspartic acid, arginine, valine and alanine in that order. Glutamine is the most abundant free amino acid in the body, comprising nearly 60% of the free intracellular amino acids in skeletal muscle. As a donor of nitrogen in the synthesis of purines and pyrimidines, glutamine is essential for the proliferation of cells. Percentage values of amino acids of all species are positively correlated with each other (*P*<0.01) and were not significantly varied with respect to various fish species (*F*_(9, 170)_ = 0.204).

**Table 3:** Amino acid composition (%) of ten fish species from Lakshadweep Sea.

Amino acid	*E. tauvina*	*C. orthogrammus*	*T. crocodilus crocodilus*	*L. gibbus*	*S. lalandi*	*T. albacares*	*P. bifasciatus*	*C. undulates*	*L. bohar*	*H. dussumieri*
**Alanine**	0.89	0.92	0.97	1.12	1.31	1.67	1.27	1.81	0.98	0.64
**Arginine**	2.31	2.55	2.87	2.53	2.55	2.51	2.33	2.41	2.37	2.47
**Asparagine**	0.76	0.54	0.81	1.92	1.55	1.84	0.96	0.91	0.86	0.77
**Aspartic acid**	0.64	0.67	0.91	0.57	0.63	0.51	0.47	0.38	0.29	0.17
**Cystine**	1.64	1.55	1.84	2.55	1.84	1.53	1.94	1.83	1.59	1.62
**Glutamic acid**	1.32	1.47	1.84	2.10	2.17	2.14	1.91	1.84	1.97	1.54
**Glutamine**	1.11	1.43	1.33	1.47	1.81	1.91	1.84	1.77	1.62	1.51
**Glycine**	0.81	0.89	1.10	1.17	1.84	1.55	0.98	0.96	0.11	0.63
**Histidine**	1.06	1.55	3.17	2.98	2.68	2.91	2.84	2.66	2.98	3.17
**Isoleucine**	3.56	4.11	3.17	3.14	2.77	2.84	3.96	3.84	4.10	2.96
**Leucine**	2.86	2.96	3.01	3.06	3.10	3.17	2.84	2.67	4.18	2.84
**Lysine**	3.17	3.19	3.81	3.82	4.13	4.56	3.98	3.61	2.84	3.96
**Methionine**	3.91	3.17	2.96	2.81	2.88	3.10	3.17	2.98	2.86	2.64
**Phenylalanine**	2.91	2.87	2.55	2.96	3.16	3.77	3.17	2.98	2.96	3.10
**Proline**	0.53	0.62	0.51	0.54	0.53	0.49	0.57	0.47	0.63	0.58
**Threonine**	1.57	2.16	1.91	1.72	1.77	1.84	1.91	1.96	1.05	2.92
**Tyrosine**	0.51	0.64	0.81	0.94	0.65	0.81	0.87	0.62	0.57	0.92
**Valine**	2.56	2.42	2.33	1.96	1.98	1.91	2.34	2.55	2.31	2.76

### 3.4 Fatty acids

Table 4 depicts the percentage as a mean value of fatty acids for each species. The fatty acid compositions of fish species ranged as follows: saturated fatty acids, SFA (31.63 to 38.97%), monounsaturated fatty acids, MUFAs (21.99–26.30%), polyunsaturated fatty acids, PUFAs (30.32–35.11%) and branched fatty acids (2.86–7.79%). Among SFAs, palmitic acid (C16:0) was the dominant saturated fatty acid (16.96%) in *E. tauvina* followed by myristic acid (C14:0) in *C. orthogrammus* (10.66%) and heneicosanoic acid (C21:0) in *H. dussumieri* (3.61%). Amounts of monounsaturated fatty acids (MUFAs) and polyunsaturated fatty acids (PUFAs) were higher in *S. lalandi* (26.30%) and *T. crocodilus crocodilus* (35.11%), respectively. The main monounsaturated fatty acid was oleic acid (C18:1ω9), accounting for 6.88% of the total fatty acids (*C. undulatus*), followed by palmitoleic acid (C16:1ω9, 6.84%) in *S. lalandi*.

**Table 4:** Fatty acid composition of ten fish species from Lakshadweep Sea.

Fatty acids (%)	*E. tauvina*	*C. orthogrammus*	*T. crocodilus crocodilus*	*L. gibbus*	*S. lalandi*	*T. albacares*	*P. bifasciatus*	*C. undulatus*	*L. bohar*	*H. dussumieri*
**Saturated Fatty acids (SFA)**										
**C10:0**	0.21	1.77	0.26	-	0.41	-	0.66	-	0.73	0.81
**C11:0**	1.98	0.84	-	0.61	0.53	0.13	-	0.11	-	0.61
**C12:0**	0.46	0.49	0.43	0.54	0.52	1.18	1.64	1.17	1.59	1.67
**C13:0**	0.11	0.39	0.11	0.16	0.10	1.64	1.51	1.79	1.83	1.54
**C14:0**	8.64	10.66	10.64	10.28	10.64	8.31	7.26	8.65	7.11	8.21
**C15:0**	0.99	1.07	1.04	1.21	1.01	2.11	2.10	2.63	2.68	2.15
**C16:0**	16.96	15.69	13.97	14.16	15.92	15.0	16.10	15.81	15.83	15.21
**C17:0**	2.55	0.51	0.64	0.53	0.53	0.68	0.54	0.51	0.67	0.59
**C18:0**	0.41	0.43	0.36	0.62	0.42	0.33	0.41	0.48	0.68	0.58
**C19:0**	2.84	0.77	0.33	1.12	0.71	-	1.11	1.61	1.57	-
**C20:0**	0.13	2.51	0.31	0.17	2.11	1.58	1.79	1.81	1.14	1.61
**C21:0**	1.11	0.64	2.21	1.19	0.20	2.61	2.34	2.55	3.11	3.61
**C22:0**	0.53	0.53	0.28	2.14	1.16	2.11	-	-	-	1.19
**C23:0**	1.21	0.54	0.51	2.55	0.17	-	0.67	-	0.68	-
**C24:0**	0.84	0.81	0.54	0.61	2.84	0.81	0.84	0.67	0.89	0.91
**Σ SFA**	38.97	37.65	31.63	35.89	37.27	36.49	36.97	38.79	38.51	38.69
**Monounsaturated fatty acids (MUSF)**										
**C14:1ω-3**	0.07	0.17	0.16	0.51	0.40	0.11	0.61	0.67	0.53	0.57
**C14:1ω-5**	0.11	-	0.81	-	0.43	0.18	-	0.16	-	0.63
**C14:1ω-7**	0.33	0.11	0.61	0.64	0.11	0.67	1.10	1.11	1.63	1.59
**C15:1ω-6**	-	0.84	0.34	-	-	1.51	1.53	0.83	0.61	0.57
**C16:1ω-5**	0.67	0.17	-	0.43	0.17	0.20	0.21	0.17	0.62	0.51
**C16:1ω-6**	0.58	-	0.51	0.32	-	0.11	0.16	0.71	0.78	0.69
**C16:1ω-7**	-	0.63	0.12	0.11	0.91	0.59	0.81	0.76	0.77	0.12
**C16:1ω-9**	5.32	4.41	5.62	6.73	6.84	4.31	3.65	4.69	4.29	4.65
**C17:1 ω-5**	0.11	-	0.22	-	0.63	1.10	-	1.17	-	1.10
**C17:1ω-7**	1.21	0.51	0.14	0.84	-	-	-	-	-	-
**C17:1ω-8**	0.89	0.63	-	0.61	0.81	0.57	-	0.63	0.57	-
**C18:1ω-5**	0.77	0.17	0.51	0.57	0.92	0.61	0.63	0.17	0.78	0.77
**C18:1ω-7**	2.11	1.74	2.16	1.75	2.17	0.81	0.83	0.63	0.55	0.59
**C18:1ω-9**	6.40	5.31	5.88	5.57	6.15	5.40	6.15	6.88	6.57	5.15
**C19:1ω-8**	0.68	-	-	-	0.16	1.11	1.05	1.17	0.87	0.53
**C20:1ω-5**	0.57	0.84	-	0.64	0.53	0.58	0.58	0.83	-	0.89
**C20:1ω-7**	0.63	-	0.62	-	-	-	1.11	-	0.29	0.71
**C20:1ω-9**	-	0.77	-	-	-	1.10	1.74	0.54	0.41	-
**C20:1ω-11**	0.11	-	-	0.52	0.41	0.33	0.21	-	-	0.23
**C22:1ω-7**	-	0.63	0.51	-	0.52	0.82	0.16	0.91	0.98	0.67
**C23:1ω-9**	0.11	0.12	1.69	0.62	0.53	0.63	0.67	0.51	0.58	0.58
**C24:1ω-3**	0.52	2.0	1.61	1.77	2.52	1.11	1.21	1.27	1.11	1.61
**C24:1ω-6**	0.17	1.0	1.62	0.53	1.11	1.63	1.14	0.55	0.54	0.64
**C24:1ω-9**	0.63	0.69	0.18	1.64	0.98	0.51	0.63	0.57	0.63	0.59
**Σ MUSF**	21.99	22.42	23.31	23.38	26.30	23.99	24.18	24.93	23.11	23.39
**Polyunsaturated fatty acids (PUFA)**										
**C16:2ω-6**	0.18	-	0.14	1.67	0.62	0.11	0.62	0.57	0.16	0.12
**C18:2ω-3**	0.22	0.63	0.18	2.62	2.11	0.19	0.86	0.81	0.84	0.67
**C18:2ω-6**	8.89	6.93	9.16	2.40	2.83	0.91	0.81	0.72	0.81	0.92
**C18:3ω-3**	1.0	1.0	1.53	1.11	1.58	5.62	4.91	3.11	4.98	4.96
**C18:3ω-6**	1.65	1.32	1.41	1.61	1.62	2.11	2.16	2.92	2.67	2.72
**C18:4ω-3**	1.62	1.43	1.63	1.82	1.91	1.41	1.43	1.01	1.63	1.57
**C19:2ω-6**	-	1.12	-	0.58	0.63	0.63	0.57	0.51	0.58	0.41
**C20:2ω-6**	1.13	1.62	1.63	1.01	0.41	1.12	1.51	1.59	1.63	1.71
**C20:3ω-6**	2.0	1.61	1.77	1.81	1.73	2.61	1.92	2.13	2.47	2.15
**C20:4ω-6**	1.68	1.98	1.11	1.32	1.57	0.16	0.17	0.18	0.51	0.66
**C20:5ω-3**	4.1	3.3	3.9	4.0	4.41	3.90	4.10	5.11	5.17	4.81
**C20:5ω-6**	2.13	1.98	1.63	1.13	1.19	1.13	1.11	1.52	1.63	1.57
**C22:3ω-3**	1.11	3.0	2.11	1.63	1.81	1.57	1.63	1.67	1.77	1.57
**C22:4ω-6**	2.0	2.1	3.2	3.0	4.92	2.11	2.17	2.63	2.13	2.60
**C22:5ω-3**	2.1	1.8	2.61	2.63	1.28	2.77	3.11	3.17	3.98	3.97
**C22:6ω-3**	2.9	2.7	3.1	2.0	2.6	4.81	4.63	2.67	2.77	2.60
**Σ PUFA**	32.71	32.52	35.11	30.34	31.22	31.96	31.51	30.32	33.73	33.01
**Σ ω-3**	13.05	13.86	15.06	15.81	15.7	20.27	20.67	17.55	21.14	20.15
**Σ ω-6**	8.94	9.29	9.34	8.27	9.82	7.13	6.88	8.05	8.37	8.69
**Σ ω-3/Σ ω-6**	1.46	1.49	1.61	1.91	1.60	2.84	3.00	2.18	2.53	2.32
**Σ ω-6/Σ ω-3**	0.69	0.67	0.62	0.52	0.63	0.35	0.33	0.46	0.40	0.43
**Branched fatty acids**										
**C11:0 Iso**	0.10	-	0.61	0.53	-	0.15	-	-	-	0.61
**C11:0 Anteiso**	-	0.11	0.57	0.41	0.17	0.16	0.16	0.11	-	0.11
**C12:0 Iso**	0.17	0.18	0.16	0.18	-	0.53	0.14	-	-	-
**C12:0 Anteiso**	0.18	0.63	0.57	0.43	0.18	-	0.11	0.12	0.16	0.12
**C13:0 Iso**	0.21	0.20	0.21	0.23	0.16	0.61	0.52	-	-	0.11
**C13:0 Anteiso**	0.18	0.17	0.62	0.51	0.15	0.53	0.48	0.22	0.18	0.17
**C14:0 Iso**	0.63	-	0.53	0.47	-	0.17	0.22	0.16	0.15	0.14
**C14:0 Anteiso**	0.18	0.62	0.81	0.56	0.16	0.19	0.17	-	0.14	-
**C15:0 Iso**	0.57	-	0.62	0.49	0.11	0.54	0.33	0.18	-	0.15
**C15:0 Anteiso**	-	0.61	0.77	0.61	0.10	0.11	0.16	0.14	0.61	-
**C16:0 Iso**	0.11	0.57	0.81	0.71	-	0.17	0.18	0.27	0.15	0.13
**C16:0 Anteiso**	-	0.11	-	0.11	0.61	0.10	0.17	-	0.36	-
**C17:0 Iso**	0.11	0.80	-	0.21	-	0.11	0.16	0.48	-	0.51
**C17:0 Anteiso**	-	0.15	0.16	0.15	0.11	0.12	0.77	0.71	0.11	0.16
**C18:0 Iso**	0.17	0.14	0.17	0.11	0.16	0.17	0.63	0.53	0.15	-
**C18:0 Anteiso**	0.13	0.11	0.14	-	0.13	0.22	0.44	0.11	0.18	0.18
**C19:0 Iso**	0.11	0.16	-	0.17	0.14	-	0.11	-	-	0.14
**C19:0 Anteiso**	0.17	-	0.16	0.90	0.17	0.10	0.51	0.16	0.18	0.13
**C20:0 Iso**	0.13	0.51	0.17	0.39	-	-	-	0.11	0.11	0.11
**C20:0 Anteiso**	0.69	0.57	0.84	0.62	0.56	0.17	0.65	0.36	0.38	0.23
**Σ Branched FA**	3.85	5.64	7.72	7.79	2.91	5.55	5.91	3.66	2.86	3.00
**Σ unidentified FA**	2.48	1.71	2.23	2.6	2.3	2.81	2.01	2.30	1.83	1.91

The dominant PUFA was linoleic acid (C18:2 ω-6) in *T. crocodilus crocodilus* accounting 9.16% of the total fatty acids, followed by alfa linolenic acid (C18:3ω-3) in *T. albacares* (5.62%), eicosapentaenoic acid (C20:5ω-3) in *L. bohar* (5.17%), docosa tetraenoic acid (C22:4ω-6) in *S. lalandi* (4.92%) and docosa hexaenoic acid (C22:6ω-3) in *T. albacares* (4.81%). Docosa hexaenoic acid (DHA) and eicosa pentaenoic acid (EPA) prevent human coronary artery diseases [29]. Therefore, fish have been suggested as a key component in the healthy diet of humans [8]. The ω-3 and ω-6 PUFAs of the ten fishes ranged 13.05–21.14% and 6.88–9.82% of the total fatty acids, respectively, and the ratio of ω-3/ω-6 was 1.46–3%, which was lower than the values reported by Zhao et al. [24] for pomfret, *P. punctatissimus*. Higher accumulation of ω-3 PUFA was observed in *L. bohar* (21.14%) and that of ω-6 PUFA was recorded in *S. lalandi* (9.82%). The ω-3/ω-6 ratio is a good index for comparing the relative nutritional values of fish oils of different species, and a higher ratio of n-3/n-6 PUFAs has often been quoted as an index of higher nutritional value. Fatty acids of all species are positively correlated with each other (*P*<0.01) and did not vary significantly with respect to various fish species (*F*_(9, 740)_ = 0.002).

Ratio of ω-6/ω-3 PUFAs (0.33–0.69%) found in the present study was lower than the value (4.0 at maximum) recommended by the UK Department of Health [30]. Values higher than the maximum are harmful to health and may promote cardiovascular diseases [9]. Differences in fatty acids of fishes are based on their diet [31], and are also affected by size, age, reproductive conditions, and environmental conditions, especially water temperature, which can influence lipid content and fatty acid composition [32]. In addition, fish provide with a favourable ratio of omega- 6 to omega-3 fatty acids. Although many omega-3 fatty acids occur in nature, DHA and EPA are not synthesized by humans at a rate to meet the metabolic needs, making a dietary source necessary [33].

Percentages of fatty acids identified differed among species and organs. In red muscle and liver, lipids undergo more enzymatic activities than in smooth muscle, producing large amounts of free fatty acids in oils [34]. Detection of higher amounts of ω-3 and ω-6 PUFA in the present fish samples is in good agreement with the findings of Huynh and Kitts [7]. The saturated and monounsaturated fatty acids are generally abundant in fish from warm or temperate regions, whereas PUFAs show higher levels in fish from cold regions [35]. Compared with freshwater fish, marine fish have higher levels of PUFAs, especially DHA and EPA. Differences in fatty acids of marine and freshwater fishes should not only be considered with respect to species habitat but also based on their natural diet, especially whether a species is herbivorous, omnivorous or carnivorous [31].

## Conclusion

Present study was carried out to find out the nutritional quality of ten economically valuable fishes of the Lakshadweep archipelago. Results showed that these fishes are a source of high quality protein, carbohydrate, lipid, ash, vitamins, with a well balanced composition of essential amino acids and fatty acids. The ω-3 and ω-6 PUFAs values were also higher in these fishes. Hence, consumption of these species is highly recommended since these fishes are more nutritious. Further, through this bio-monitoring study, present results of the proximate composition analysis, amino acid and fatty acid analysis can be used as baseline data for comparisons in future, with regard to fish nutritional quality.
